# Carotenoid Metabolism in Plant Stress Adaptation: Pathway Regulation, Flux Distribution, and Stress-Adaptive Functions

**DOI:** 10.3390/plants15213383

**Published:** 2026-11-01

**Authors:** Panrui Chen, Zhengyao Lin, Mingjian Zhou, Jie Shen, Yinggao Liu, Fuyuan Zhu, Yanjie Xie

**Affiliations:** National Key Laboratory for the Development and Utilization of Forest Food Resources, Co-Innovation Center for Sustainable Forestry in Southern China, State Key Laboratory of Tree Genetics and Breeding, Key Laboratory of State Forestry and Grassland Administration on Subtropical Forest Biodiversity Conservation, College of Life Sciences, Nanjing Forestry University, Nanjing 210037, China

**Keywords:** carotenoids, metabolic pathway, carotenoid flux, stress response, ABA, resistance mechanism, regulatory network

## Abstract

Carotenoids are plastid-localized isoprenoid metabolites essential for photosynthesis, photoprotection, and plant development. Their metabolism also provides precursors for abscisic acid (ABA), strigolactones (SLs), and diverse apocarotenoids involved in environmental adaptation. Both abiotic and biotic stresses can alter carotenoid biosynthesis, composition, and turnover, although the underlying regulatory mechanisms and physiological consequences vary with stress type. This review examines how carotenoid pathway regulation influences metabolite allocation and stress-adaptive functions, with particular emphasis on the control of precursor supply and metabolic branch points. We discuss the contributions of carotenoid metabolism to ABA-mediated stress responses, photoprotection and redox homeostasis, and signaling by carotenoid-derived metabolites, including zaxinone, β-ionone, β-cyclocitral, and SLs. The distinct and overlapping roles of these processes in abiotic stress adaptation and biotic defense are evaluated, while senescence is considered as a developmental process associated with changes in carotenoid turnover and downstream signaling. We further examine how functional specialization among pathway genes, transcriptional and chromatin regulation, RNA-mediated control, protein stability, and enzyme interactions influence pathway activity. Metabolite feedback and competition for shared precursors, particularly at the methylerythritol 4-phosphate (MEP) and geranylgeranyl diphosphate (GGPP) nodes, provide additional mechanisms for regulating metabolic branch allocation. The reversible interconversion between ABA and ABA glucose ester (ABA-GE) is also discussed as a mechanism of ABA homeostasis distinct from de novo carotenoid-dependent biosynthesis. Importantly, the extent to which changes in metabolite abundance reflect actual flux redistribution remains insufficiently resolved. Future research should therefore prioritize quantitative flux analysis, the mechanisms underlying tissue- and stress-specific regulation, the perception of carotenoid-derived signals, and strategies to improve stress resilience while minimizing growth and yield penalties.

## 1. Introduction

Carotenoids are plastid-localized isoprenoid metabolites that contribute to light harvesting, photosynthesis, photoprotection, and plant development [1]. They are also metabolic precursors of abscisic acid (ABA), strigolactones (SLs), and several other apocarotenoids [2]. Environmental stress can change carotenoid synthesis and turnover, with effects on photosynthetic protection, cellular redox balance, and the production of signaling-active metabolites [3]. For example, the xanthophyll cycle contributes to the dissipation of excess excitation energy, whereas carotenoid cleavage and oxidation generate compounds involved in hormonal and defense responses [4]. These functions connect plastidial metabolism with environmental adaptation, although the relevant metabolic outputs differ among biological conditions.

Carotenoid pathway genes have been characterized in several plant species, including Arabidopsis (*Arabidopsis thaliana*), tomato (*Solanum lycopersicum*), and maize (*Zea mays*) [5]. Changes in carotenoid content and composition under stress indicate that pathway activity responds to environmental conditions [6]. Abiotic challenges often create strong demands for photoprotection and ABA-associated responses, whereas pathogen and herbivore challenges can involve carotenoid-derived volatiles and defense signaling. These are overlapping tendencies rather than mutually exclusive pathways: reactive oxygen species (ROS), hormones, and apocarotenoids participate in both types of response [7,8,9]. However, the evidence remains distributed across species, tissues, and experimental conditions. In particular, how individual pathway genes cooperate or compensate for one another, how members of multigene families acquire distinct stress-responsive functions, and how metabolic flux is redistributed among competing branches remain insufficiently understood. A pathway-wide view integrating metabolic regulation with stress signaling is therefore needed.

This review first introduces the principal carotenoid biosynthetic and cleavage steps, followed by their contributions to ABA-mediated responses, photoprotection, antioxidant defense, and carotenoid-derived signaling. We then compare the evidence obtained under abiotic and biotic stresses. Senescence is discussed separately as a developmental process involving chloroplast remodeling, carotenoid turnover, and hormone-associated changes. Subsequent sections examine gene specialization and multilevel regulation, followed by metabolite feedback and the allocation of shared isoprenoid precursors. We also consider reversible ABA–ABA-GE interconversion as part of active ABA homeostasis. Finally, we discuss the evidence needed to distinguish pathway reorganization from quantitative flux changes and to guide stress-resilience engineering without excessive growth costs.

## 2. Carotenoid Metabolism: Pathway Architecture and Regulatory Nodes

Carotenoid biosynthesis occurs in plastids and is supplied by the methylerythritol 4-phosphate (MEP) pathway. This pathway generates the isoprenoid precursors isopentenyl diphosphate (IPP) and dimethylallyl diphosphate (DMAPP) [10]. Geranylgeranyl diphosphate synthase (GGPPS) uses these precursors to produce geranylgeranyl diphosphate (GGPP). Phytoene synthase (PSY) then condenses two GGPP molecules to form 15-*cis*-phytoene, representing the first committed step of carotenoid biosynthesis and an important control point for pathway flux [11].

Phytoene is subsequently converted to all-*trans*-lycopene through sequential reactions catalyzed by phytoene desaturase (PDS), ζ-carotene isomerase (Z-ISO), ζ-carotene desaturase (ZDS), and carotenoid isomerase (CRTISO) [12]. Lycopene represents a major branch point in the pathway. Lycopene β-cyclase (LCYB), acting alone or with lycopene ε-cyclase (LCYE), contributes to the formation of β- and α-carotene, respectively. Subsequent hydroxylation and related reactions produce xanthophylls, including zeaxanthin and lutein. Within the xanthophyll cycle, zeaxanthin epoxidase (ZEP) and violaxanthin de-epoxidase (VDE) mediate the reversible interconversion between zeaxanthin and violaxanthin. This cycle is particularly important for photoprotection under fluctuating environmental conditions [13].

Beyond pigment biosynthesis, carotenoid cleavage connects pathway flux with stress signaling. 9-*cis*-epoxycarotenoid dioxygenase (NCED)-mediated cleavage of epoxycarotenoids provides the entry point to ABA biosynthesis, whereas other carotenoid cleavage dioxygenases (CCDs) generate SLs and diverse apocarotenoids involved in development and environmental responses [14,15]. Zaxinone synthase (ZAS) generates another signaling-active apocarotenoid, zaxinone [4]. Carotenoid metabolism therefore comprises several interconnected control points, from precursor supply and branch partitioning to xanthophyll cycling and carotenoid cleavage. This pathway architecture provides the metabolic basis for the photoprotective, hormonal, and signaling functions discussed in the following sections.

## 3. Carotenoid Metabolism in Stress Adaptation

Environmental stresses perturb photosynthetic activity, cellular redox homeostasis, and carbon allocation, thereby creating a strong demand for metabolic adjustment [16]. Carotenoid metabolism is therefore dynamically remodeled rather than functioning as a static pigment-producing pathway. Changes in biosynthetic activity and turnover affect carotenoid abundance and composition and can modify the availability of precursors for downstream signaling molecules [17]. These metabolic shifts contribute to stress adaptation through several interconnected outputs, including ABA-mediated responses, photoprotection and antioxidant defense, and the generation of carotenoid-derived signals [18]. Because these outputs originate from a shared metabolic network, changes at one pathway node can influence multiple downstream processes simultaneously. Understanding carotenoid responses therefore requires examining individual metabolites and enzymes alongside the mechanisms that may alter substrate allocation among pathway branches. The following sections focus on three major functional dimensions of this reprogramming: ABA-related stress responses, photoprotective and redox regulation, and carotenoid-derived signaling.

### 3.1. Carotenoid Flux and ABA-Mediated Stress Adaptation

**Figure 1:**
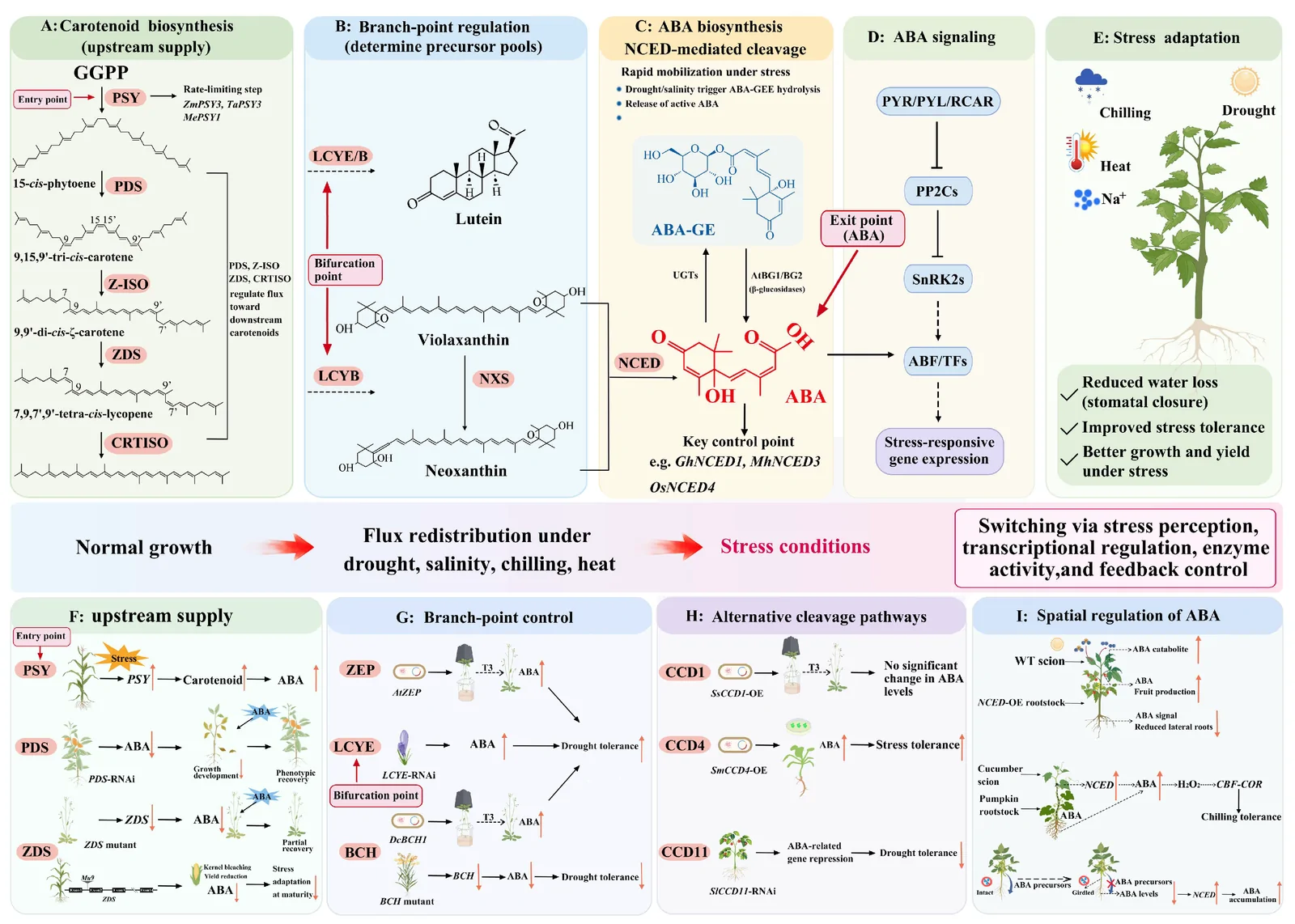
Carotenoid flux control of ABA biosynthesis and stress adaptation. Carotenoid-derived precursor supply, pathway partitioning, and spatial regulation collectively determine ABA biosynthetic capacity and ABA-mediated stress responses. Legend for the carotenoid flux framework: Red boxes/labels and red arrows mark key elements. Entry point: PSY-mediated entry of GGPP into carotenoid biosynthesis (**A**,**F**). Branch point: LCYE/LCYB partitions flux toward the lutein and β-carotene/ABA branches (**B**,**G**). Exit point: ABA output to ABA signaling (**C**). Key control point: NCED-mediated oxidative cleavage, a major node in stress regulation (**C**). Meanwhile, this node involves the dynamic mobilization mechanism of ABA-GE: UGTs catalyze ABA to form the inactive storage form ABA-GE; under drought/salinity stress, AtBG1/BG2 (β-glucosidases) rapidly hydrolyze ABA-GE to release active ABA for rapid response to environmental changes. The central panel shows environmental contexts (drought, salinity, chilling, and heat) and switching mechanisms (stress perception, transcriptional regulation, enzyme activity, and feedback control). (**A**) Upstream carotenoid biosynthesis supplies substrates for ABA production. Phytoene synthase (PSY) controls the committed entry of geranylgeranyl diphosphate (GGPP) into carotenoid biosynthesis, followed by sequential desaturation and isomerization reactions mediated by phytoene desaturase (PDS), ζ-carotene isomerase (Z-ISO), ζ-carotene desaturase (ZDS), and carotenoid isomerase (CRTISO). (**B**) Lycopene cyclization and subsequent xanthophyll metabolism determine the abundance of downstream carotenoid pools, including violaxanthin and neoxanthin, that provide substrates for ABA biosynthesis. (**C**) 9-*cis*-Epoxycarotenoid dioxygenases (NCEDs) catalyze the oxidative cleavage of 9-*cis*-epoxycarotenoids to produce xanthoxin, a major regulatory step in ABA biosynthesis. (**D**) ABA is perceived by PYR/PYL/RCAR receptors, leading to inhibition of type 2C protein phosphatases (PP2Cs), activation of SnRK2 protein kinases, and downstream regulation of ABA-responsive transcription factors and gene expression. (**E**) ABA-dependent responses contribute to stomatal closure, water conservation, and adaptation to drought, salinity, chilling, and heat stress. (**F**) Representative studies illustrate how modulation of upstream enzymes, including PSY, PDS, and ZDS, alters carotenoid precursor supply and ABA accumulation. (**G**) Regulation at branch-point and xanthophyll-cycle enzymes, including LCYE, BCH, and ZEP, further modifies the availability of ABA precursors and stress responses. (**H**) Carotenoid cleavage dioxygenases outside the canonical NCED pathway can also influence ABA accumulation or ABA-associated stress responses in a species-dependent manner. (**I**) ABA responses are further shaped by the spatial regulation of ABA biosynthesis and transport among roots, shoots, and graft partners. ABA, abscisic acid; BCH, β-carotene hydroxylase; CCD, carotenoid cleavage dioxygenase; CRTISO, carotenoid isomerase; GGPP, geranylgeranyl diphosphate; LCYE, lycopene ε-cyclase; NCED, 9-*cis*-epoxycarotenoid dioxygenase; PDS, phytoene desaturase; PSY, phytoene synthase; ZDS, ζ-carotene desaturase; and ZEP, zeaxanthin epoxidase. Arrows in the figure represent positive regulation, while blunt-ended lines represent negative regulation.

In the carotenoid pathway, ABA biosynthesis provides the most direct connection between pathway flux and hormonal regulation [19]. ABA production depends on the availability of carotenoid-derived precursors and their subsequent cleavage by NCEDs. NCEDs catalyze cleavage of 9-*cis*-violaxanthin and 9-*cis*-neoxanthin to produce xanthoxin and therefore represent an important control point in ABA biosynthesis [20] (Figure 1A–C). Genetic studies across several plant species support this role. Silencing *GhNCED1* in cotton (*Gossypium hirsutum*) reduces ABA accumulation and compromises drought and salt tolerance [21], whereas heterologous expression of *MhNCED3* or *OsNCED4* restores ABA levels and drought tolerance in the *Arabidopsis nced3* mutant [22,23]. Mutations in several rice (*Oryza sativa* L.) *NCED* genes similarly reduce ABA accumulation and increase sensitivity to abiotic stress [24]. These studies link NCED-dependent cleavage with ABA accumulation and associated stress responses (Figure 1C).

However, ABA production is not determined by NCED activity alone. The amount of substrate available for cleavage also depends on upstream carotenoid biosynthesis [25] (Figure 1A–C). *PSY* catalyzes the first committed step of the pathway and can constrain downstream flux under specific physiological conditions. In maize, stress-induced *ZmPSY3* expression in roots contributes to ABA accumulation [26] (Figure 1F), whereas *TaPSY3* promotes ABA biosynthesis and is associated with improved drought and heat tolerance in wheat (*Triticum aestivum* L.) [27]. Cassava (*Manihot esculenta*) further illustrates the importance of tissue context. *MePSY1* contributes to ABA production in leaves, but its contribution to carotenoid production is more evident in roots, where carotenoid abundance is relatively low, whereas *NCED* remains a major control point in leaves [28]. The relative contribution of precursor supply and NCED-mediated cleavage to ABA production can therefore vary among tissues and environmental conditions.

Downstream carotenoid biosynthesis further shapes the pool of ABA precursors. *PDS*, *Z-ISO*, *ZDS*, and *CRTISO* regulate the conversion of phytoene toward more highly desaturated carotenoids and thereby influence substrate availability for ABA synthesis [29]. In citrus (*Citrus sinensis* L.), *PDS* silencing reduces zeaxanthin and ABA accumulation [30], whereas the *Arabidopsis spc1-1* (*ZDS*) mutant shows ABA deficiency [31]. More severe disruption of *ZDS* also affects chloroplast development, indicating that reduced carotenoid flux can influence both ABA biosynthesis and plastid function. In maize, *ZDS* disruption similarly lowers ABA accumulation and contributes to vivipary and yield loss [32] (Figure 1F).

Branch-point reactions provide an additional level of control by altering the relative abundance of carotenoid pools entering the ABA pathway (Figure 1B). Suppression of *CstLCYE* in saffron (*Crocus sativus* L.) increases ABA accumulation and abiotic stress tolerance [33], whereas overexpression of *DcBCH1*, encoding β-carotene hydroxylase (BCH1), increases ABA levels and drought tolerance in *Arabidopsis* [34]. By contrast, disruption of rice *BCH* reduces zeaxanthin and ABA accumulation and compromises drought tolerance [35]. *ZEP* is positioned at the intersection of the xanthophyll cycle and ABA biosynthesis, but its contribution is also species- and tissue-dependent [36] Altering *ZEP* expression has little effect on ABA accumulation in tobacco (*Nicotiana tabacum* L.), whereas *AtZEP* overexpression enhances ABA synthesis and drought tolerance in *Arabidopsis*, with different responses in roots and leaves [37] (Figure 1G).

Carotenoid cleavage pathways outside the canonical ABA route can also influence ABA-related responses by altering shared substrate pools or interacting with other signaling pathways. Individual CCD family members have been associated with changes in ABA accumulation and stress tolerance, although these effects vary among species [38]. *SsCCD1* overexpression does not increase ABA in *Arabidopsis* [39], whereas *SmCCD4* promotes ABA accumulation in *Salvia miltiorrhiza* [40], and *SlCCD11* contributes to salt tolerance in tomato [41] (Figure 1H). The physiological effect of ABA also depends on where it is produced and redistributed. In tomato, NCED-overexpressing rootstocks improve salt tolerance and yield while altering ABA distribution among roots, leaves, and reproductive tissues [42]. In cucumber (*Cucumis sativus*)–pumpkin (*Cucurbita maxima*) grafts, rootstock-induced NCED expression and ABA transport contribute to chilling tolerance through signaling involving H_2_O_2_, C-repeat binding factors (CBFs), and cold-regulated (COR) genes [43]. In soybean (*Glycine max*), root ABA accumulation during dehydration appears to depend mainly on local *NCED* induction rather than shoot-to-root ABA transport [44] (Figure 1I). The above examples indicate that ABA production initiates stress-responsive signaling, ultimately manifesting as macroscopic stress-adaptive phenotypes in which plants conserve water and maintain growth under various abiotic stresses (Figure 1D,E).

Active ABA levels also depend on reversible conjugation independently of de novo carotenoid cleavage. UDP-glucosyltransferases (UGTs) convert ABA into ABA glucose ester (ABA-GE), a conjugated form associated with hormone storage and transport [45,46]. β-Glucosidases, including AtBG1 and AtBG2, can hydrolyze ABA-GE to release active ABA [47,48]. Mobilization of this pool during dehydration and related stress conditions can contribute to rapid changes in ABA availability, with consequences for stomatal responses and stress-responsive gene expression [49,50,51]. ABA biosynthetic flux and ABA-GE turnover should therefore be distinguished when interpreting changes in the active hormone pool (Figure 1C).

### 3.2. Photoprotection, Redox Buffering, and Antioxidant Defense

Environmental stress often disrupts photosynthetic electron transport and promotes the accumulation of ROS. Carotenoids limit such damage through energy dissipation, direct quenching of reactive species, and stabilization of photosynthetic membranes [52]. The xanthophyll cycle is particularly important in this process. VDE converts violaxanthin to zeaxanthin under excess excitation, whereas ZEP catalyzes the reverse reaction [53]. In addition to its role in non-photochemical quenching (NPQ), zeaxanthin can quench singlet oxygen and other reactive species and restrict lipid peroxidation [54]. Consistent with these protective functions, exogenous zeaxanthin reduces chilling injury in pepper (*Capsicum annuum* L.) by improving photosynthetic performance and limiting ROS accumulation [55].

Genetic manipulation of xanthophyll metabolism further supports its role in photoprotection. Reduced VDE activity in the *Arabidopsis eto1-1* and *ctr1-3* mutants is associated with lower zeaxanthin accumulation, higher ROS levels, and impaired growth, whereas increased VDE activity partially alleviates these phenotypes [56]. Antisense suppression of *VDE*, encoding violaxanthin de-epoxidase, in tobacco similarly reduces xanthophyll de-epoxidation and NPQ and aggravates high-light damage [57]. By contrast, overexpression of *BCH*, which encodes β-carotene hydroxylase, increases zeaxanthin accumulation in *Arabidopsis* and improves tolerance to high light and low temperature. This protection is attributed not only to thermal energy dissipation but also to the antioxidant and membrane-protective properties of zeaxanthin [58]. Carotenoid turnover can also modify the availability of photoprotective xanthophylls. In saffron, developmental changes in *CsCCD2L*, encoding a carotenoid cleavage dioxygenase 2-like protein, are associated with changes in zeaxanthin abundance and photooxidative tolerance [59].

Beyond the xanthophyll cycle, upstream carotenoid biosynthetic steps also shape the size and composition of the pigment pool available for photoprotection and redox buffering. In rice, disruption of *ZDS*, encoding ζ-carotene desaturase, or *CRTISO*, encoding carotenoid isomerase, causes excessive ROS accumulation together with severe developmental defects or reduced heat tolerance [60,61]. In cotton, suppression of *LCYE*, which encodes lycopene ε-cyclase, reduces lutein accumulation and compromises drought tolerance [62]. Conversely, increased expression of *LCYB* and *LCYE*, encoding lycopene β-cyclase and lycopene ε-cyclase, respectively, can enhance carotenoid accumulation and ROS-scavenging capacity [63]. Targeted disruption of tomato *LCYB* increases lycopene accumulation and improves postharvest chilling tolerance [64]. These observations indicate that stress protection depends not only on total carotenoid abundance but also on how pathway flux is distributed among carotenoid species with different photoprotective and antioxidant properties.

Changes in carotenoid metabolism are also frequently accompanied by adjustments in the enzymatic antioxidant system. Enhanced expression of *IbZDS*, encoding ζ-carotene desaturase, and of *AhNCED4* or *MaNCED1*, encoding 9-*cis*-epoxycarotenoid dioxygenases, has been associated with higher activities of superoxide dismutase (SOD), peroxidase (POD), and catalase (CAT), together with increased proline accumulation and lower malondialdehyde (MDA) and H_2_O_2_ levels under stress [65,66,67,68]. Conversely, silencing of *CaLCYB*, encoding lycopene β-cyclase, is associated with reduced antioxidant enzyme activities, enhanced lipid peroxidation, and lower stress tolerance [69]. These associations suggest that changes in carotenoid metabolism can influence cellular redox status beyond the direct antioxidant activity of carotenoid molecules themselves.

The mechanistic connection between carotenoid metabolism and the enzymatic antioxidant system remains unresolved. Increased activities of SOD, POD, or CAT after manipulation of carotenoid-pathway genes do not necessarily indicate that carotenoids directly activate antioxidant defense. These responses may instead result from altered ABA accumulation, carotenoid-derived signaling molecules, changes in chloroplast redox status, or improved photosynthetic performance. Clarifying these mechanisms is important for distinguishing the direct photoprotective and antioxidant functions of carotenoids from the signaling activities of their metabolic derivatives. The latter become particularly relevant when carotenoid turnover generates bioactive compounds that participate in stress signaling.

### 3.3. Carotenoid-Derived Signals in Stress Adaptation

Carotenoid cleavage and oxidation generate compounds with signaling activities that extend beyond the functions of intact pigments. These metabolites influence root development, defense, plastid-to-nucleus communication, and interactions among plants and with their surrounding organisms. Beyond ABA, the main examples considered here are zaxinone, β-ionone, β-cyclocitral (β-CC), and SLs. Their biosynthetic origins and physiological effects differ, and evidence for direct signal perception remains stronger for some compounds than for others [70].

#### 3.3.1. Zaxinone

**Figure 2:**
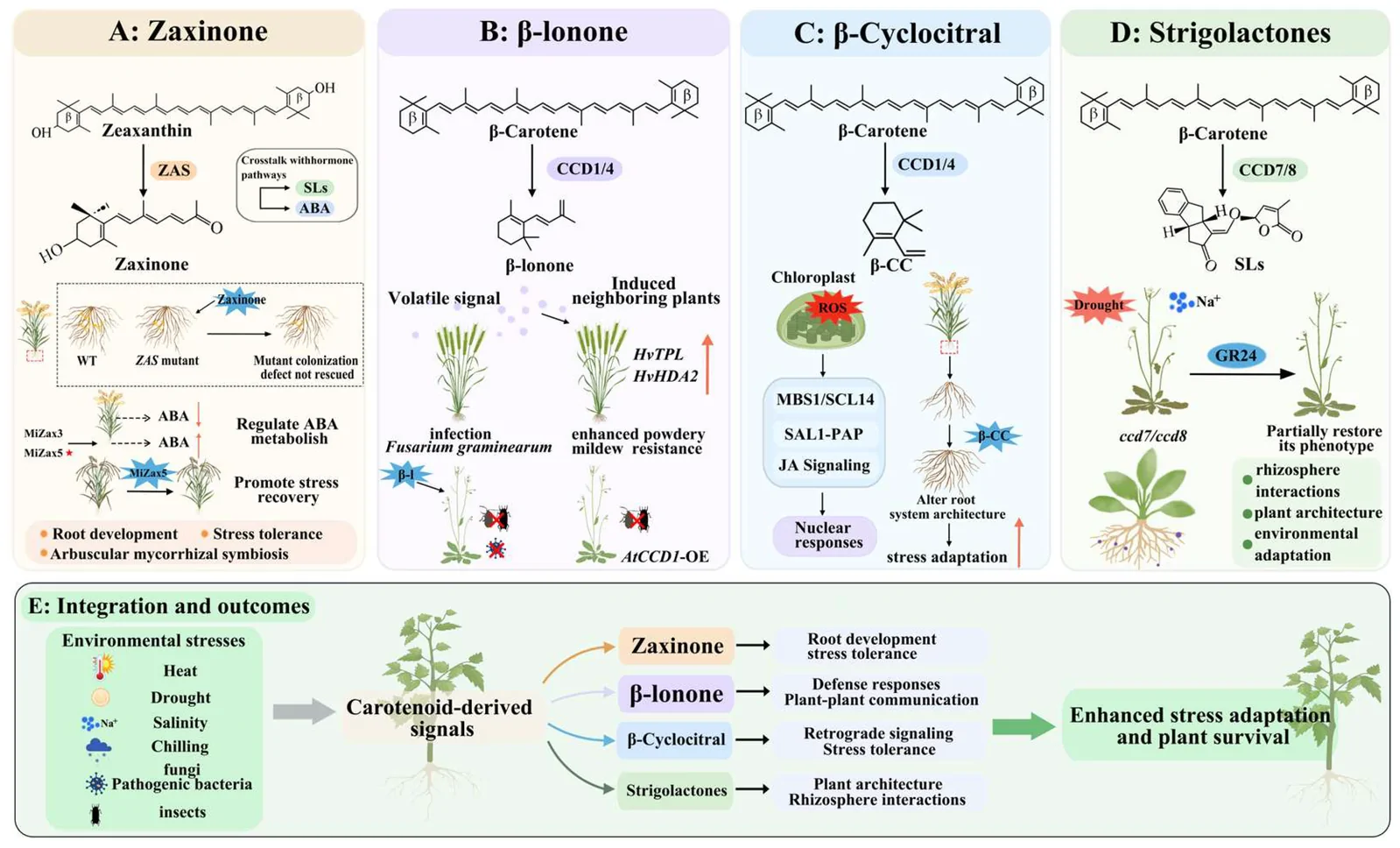
Carotenoid-derived signals in plant stress adaptation. Carotenoid cleavage and oxidation generate structurally distinct signaling-active metabolites that connect carotenoid metabolism with hormone regulation, root development, defense, rhizosphere interactions, and plastid-to-nucleus signaling. (**A**) Zaxinone. Zaxinone synthase (ZAS) converts zeaxanthin into zaxinone. Zaxinone influences root development and interacts with ABA and strigolactone (SL) metabolism, while ZAS-dependent processes are also associated with arbuscular mycorrhizal symbiosis and drought adaptation. Synthetic zaxinone analogs such as MiZax5 can modify ABA metabolism and promote stress recovery. The asterisk in the figure indicates that the synthetic zaxinone analog MiZax5 promotes post-stress recovery more effectively than MiZax3. (**B**) β-Ionone. β-Ionone is a volatile carotenoid-derived metabolite involved in pathogen and herbivore defense. Its production can influence local resistance and, through volatile emission, activate defense responses in neighboring plants. (**C**) β-Cyclocitral. β-Cyclocitral (β-CC) is generated from β-carotene under oxidative conditions and functions in chloroplast-derived stress signaling. β-CC regulates root architecture and promotes nuclear stress responses through pathways involving MBS1/SCL14, SAL1–PAP, and hormone-associated signaling, thereby linking carotenoid oxidation with cellular redox status and environmental adaptation. (**D**) Strigolactones. SL biosynthesis originates from β-carotene and involves carotenoid isomerization and sequential cleavage reactions mediated by D27, CCD7, and CCD8. SLs regulate plant architecture, rhizosphere interactions, and responses to drought and salinity; the synthetic SL analog GR24 can partially complement stress-sensitive phenotypes of SL-deficient mutants. (**E**) These carotenoid-derived signals exert distinct but interconnected functions in root adaptation, defense communication, retrograde signaling, plant architecture, and rhizosphere interactions, thereby contributing to plant survival under environmental stress. ABA, abscisic acid; β-CC, β-cyclocitral; CCD, carotenoid cleavage dioxygenase; D27, DWARF27; MBS1, METHYLENE BLUE SENSITIVITY 1; PAP, 3′-phosphoadenosine 5′-phosphate; SAL1, SAL1 phosphatase; SLs, strigolactones; and ZAS, zaxinone synthase. In the figure, solid arrows indicate positive regulation, promotion, or metabolic flow, whereas blunt-ended lines indicate negative regulation or inhibition.

Zaxinone is a carotenoid-derived metabolite produced from zeaxanthin by ZAS. It links carotenoid metabolism with root development, drought responses, and rhizosphere interactions [38]. In rice, zaxinone modulates SLs metabolism and contributes to drought adaptation, while its synthetic analog MiZax5 improves stem water potential, alters ABA metabolism, and promotes recovery from stress [71]. In *Arabidopsis*, exogenous zaxinone affects both SL and ABA biosynthesis and inhibits hypocotyl growth [72]. Zaxinone is also associated with arbuscular mycorrhizal symbiosis. Rice *ZAS* mutants show reduced mycorrhizal colonization, but exogenous zaxinone does not fully restore this phenotype, suggesting that ZAS-dependent regulation may involve additional processes beyond zaxinone itself [73] (Figure 2A,E). These findings indicate that zaxinone participates in stress adaptation through coordinated effects on hormone metabolism, root development, and plant-microbe interactions.

#### 3.3.2. β-Ionone

β-Ionone is a volatile carotenoid cleavage product with prominent roles in plant defense and interplant communication, particularly under biotic stress. Exogenous β-ionone enhances resistance to Botrytis cinerea in *Arabidopsis*, and similar protective effects have been reported in tobacco and tomato [7]. β-Ionone also affects plant–insect interactions by repelling herbivores or reducing oviposition. Increased β-ionone production following *AtCCD1* overexpression reduces flea beetle feeding [74,75]. Its signaling activity can extend beyond the producing tissue or plant. In barley infected with *Pseudomonas syringae* pv. japonica, released β-ionone induces defense-associated genes, including *HvHDA2* and *HvTPL*, in neighboring plants and enhances resistance to *Blumeria graminis* [8] (Figure 2B,E). β-Ionone therefore links carotenoid turnover with both local defense responses and volatile-mediated communication between plants under biotic stress.

#### 3.3.3. β-Cyclocitral (β-CC)

β-CC provides a particularly clear example of how carotenoid oxidation can generate a retrograde stress signal. β-CC is produced from β-carotene during oxidative stress and participates in plastid-to-nucleus communication [76]. Increased β-CC production enhances tolerance to dehydration, salinity, and oxidative stress, whereas exogenous β-CC can modify root architecture and stress physiology in rice [77]. Its oxidation product β-cyclocitric acid (β-CCA) further contributes to drought adaptation by promoting drought-responsive transcription and root suberization. β-CC and β-CCA also exert distinct effects on root development, indicating that closely related carotenoid-derived metabolites can produce different physiological outputs [78]. Under high light, β-CC acts downstream of singlet oxygen (^1^O_2_) and promotes nuclear stress responses through METHYLENE BLUE SENSITIVITY 1 (MBS1)/SCARECROW-LIKE 14 (SCL14), the SAL1 phosphatase-3′-phosphoadenosine 5′-phosphate (PAP) pathway, and salicylic acid-associated signaling [9,79,80] (Figure 2C,E). β-CC thus links carotenoid oxidation with chloroplast redox status and downstream transcriptional regulation.

#### 3.3.4. Strigolactones (SLs)

SLs are carotenoid-derived hormones that connect carotenoid metabolism with plant architecture, rhizosphere interactions, and environmental adaptation [81]. Their biosynthesis involves sequential carotenoid cleavage reactions mediated by *CCD7* and *CCD8*, leading toward the formation of carlactone and downstream SLs [82]. *Arabidopsis ccd7*/*ccd8* mutants are hypersensitive to drought and salinity, and treatment with the synthetic strigolactone analog GR24 partially rescues these phenotypes [83]. SL biosynthesis is itself responsive to environmental and hormonal signals. In apple (*Malus domestica*), stress and methyl jasmonate suppress *MdCCD7* expression, with *MdBPC1* contributing to this regulation [84]. In rice, D27 further links SL metabolism with ABA homeostasis during drought, as changes in D27 activity are associated with altered ABA accumulation and drought responses [85] (Figure 2D,E). SL biosynthesis uses carotenoid-derived precursor pools that also support other metabolic branches. Changes in precursor supply may therefore affect SL production alongside other signaling outputs. Differences in the production and action of these compounds highlight the importance of tissue- and condition-specific pathway regulation.

### 3.4. Carotenoid Metabolism Under Abiotic and Biotic Stresses

Abiotic and biotic stresses place different demands on carotenoid metabolism, although many of the underlying responses overlap. Drought, salinity, temperature extremes, and excess light commonly place demands on photoprotection, redox balance, and ABA-associated responses. Pathogen infection and herbivory can additionally involve defense hormones and the production or emission of carotenoid-derived compounds, particularly volatile β-ionone. These processes overlap; however, ROS and hormonal signaling can influence carotenoid metabolism during either type of stress, and β-cyclocitral should not be regarded as a biotic-stress-specific product. The direction of pathway changes is likely to depend on the interacting stresses, plant species, tissue, and developmental stage.

Consistent with this context dependence, evidence for context-specific responses comes from combined water stress and Alternaria dauci infection in carrot, where carotenoid contents differed among organs and genotypes [86]. During herbivore attack, lettuce accumulates β-ionone and β-cyclocitral, with LsCCD1 implicated in β-ionone production through β-carotene cleavage [87]. β-Cyclocitral has also been implicated in feedback regulation of plastidial isoprenoid metabolism [88]. Together, these studies support changes in carotenoid pools or signaling outputs, but they do not establish a universal shift in metabolic flux between abiotic and biotic stresses. Comparisons using matched tissues, stress time courses, and quantitative flux analyses will be needed to distinguish shared responses from stress-specific mechanisms. Beyond stress, senescence offers a further developmental comparison because pigment maintenance, carotenoid turnover, and hormone-associated processes change as chloroplasts are dismantled.

## 4. Carotenoid Metabolism During Senescence

**Figure 3:**
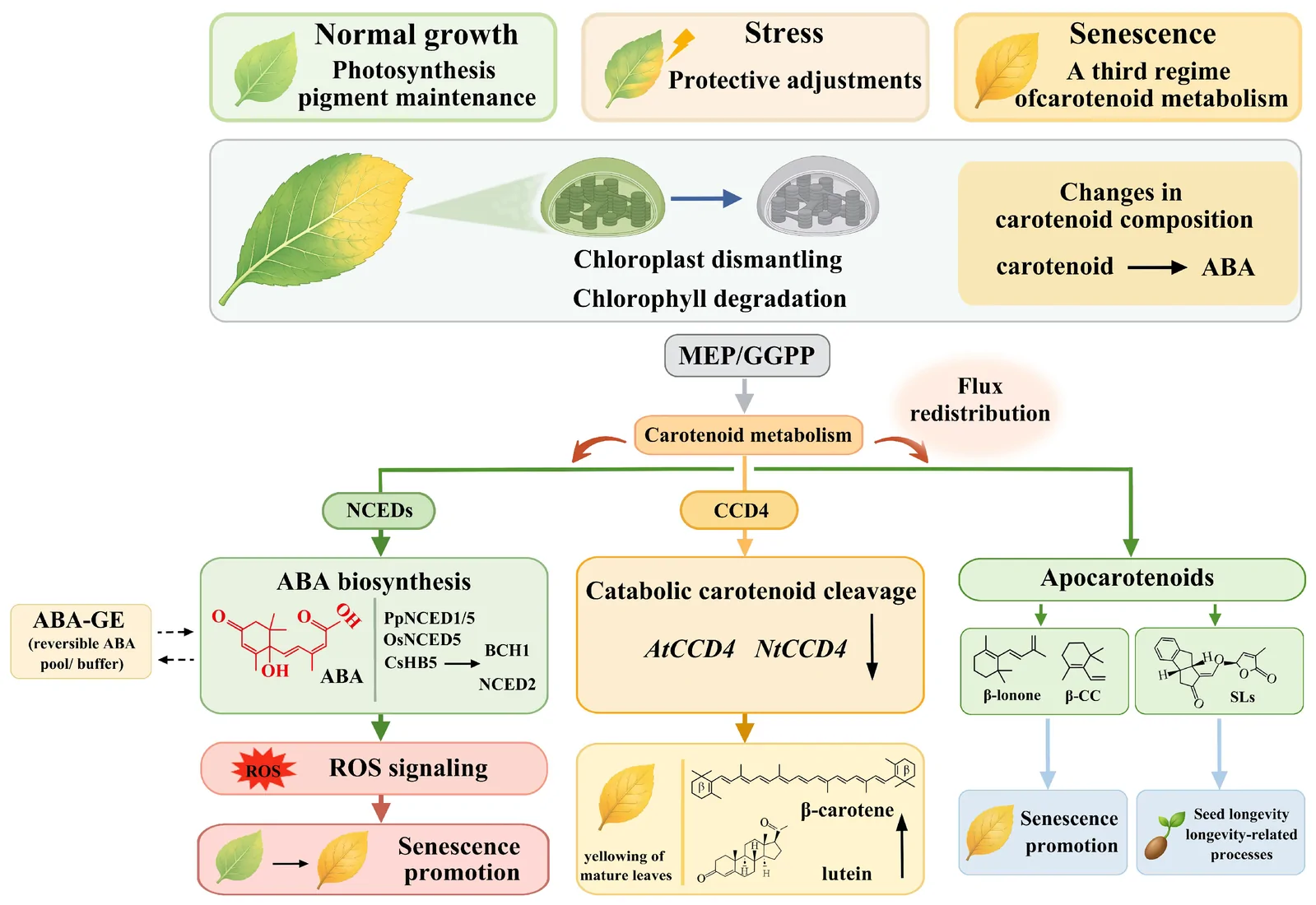
Senescence as a third regime of carotenoid metabolism. During senescence, chloroplast dismantling and chlorophyll degradation occur, and carotenoid flux is thought to be redistributed through the MEP/GGPP node, potentially shifting from photosynthetic pigment maintenance toward ABA biosynthesis, CCD4-mediated cleavage, and apocarotenoid signaling. NCEDs are associated with promoting ABA accumulation and ROS signaling, accelerating senescence; CCD4 contributes to carotenoid degradation and leaf yellowing; SLs and β-ionone, among others, have been implicated in the regulation of senescence and seed longevity. Normal growth, stress, and senescence collectively can be conceptualized as part of a three-regime framework for carotenoid metabolism. In the figure, solid arrows indicate positive regulation, promotion, or metabolic flow.

Senescence provides a developmental setting in which the functions of carotenoid metabolism change as photosynthetic structures are dismantled. During leaf senescence, chloroplast remodeling and chlorophyll degradation are accompanied by changes in carotenoid composition and turnover [3]. Carotenoid cleavage can also contribute to ABA-associated developmental responses. In peach (*Prunus persica*) fruit, *PpNCED1* and *PpNCED5* contribute to ABA accumulation during ripening and senescence-associated processes, with reported links to reactive oxygen species (ROS) signaling [89]. Rice *OsNCED5* participates in drought and salt responses as well as leaf senescence [24]. The citrus transcription factor *CsHB5* also regulates carotenoid-pathway genes, including *BCH1* and *NCED2*, and promotes senescence-related phenotypes in heterologous systems [90] (Figure 3). These examples implicate specific carotenoid metabolic nodes in senescence, although changes in enzyme expression or product abundance alone do not quantify pathway flux (Figure 3).

Carotenoid cleavage can become prominent as chloroplast functions decline and photosynthetic pigment pools are remodeled. In *Arabidopsis*, *CCD4* contributes to carotenoid degradation during dark-induced leaf senescence, and the ccd4 mutant shows altered β-carotene accumulation [91]. Loss of *NtCCD4* function in tobacco also affects leaf coloration and carotenoid content during maturation [92]. Hormonal products derived from carotenoids can have separate roles in leaf aging: strigolactone-deficient or -insensitive mutants of *Arabidopsis* and rice display delayed leaf senescence, whereas exogenous GR24 can promote senescence in strigolactone-deficient backgrounds [93]. β-Cyclocitral and β-ionone have also been associated with improved seed longevity [94] (Figure 3).

Thus, senescence provides a natural developmental context in which the carotenoid pathway is thought to undergo regime switching and possible flux redistribution. During this transition, precursor allocation at the MEP/GGPP nodes is predicted to shifts from photosynthetic pigment maintenance toward ABA, SLs, and apocarotenoid production, while reversible ABA pools such as ABA-GE can buffer active ABA concentrations and serve as a rapidly mobilizable source under changing developmental conditions. Together with normal growth and stress, senescence can be conceptualized as part of a three-regime conceptual framework for carotenoid flux, in which the same pathway operates under different metabolic priorities through changes in entry points, bifurcation points, exit points, and regulatory inputs.

## 5. Multilevel Regulation of Carotenoid Pathway Activity

The different carotenoid-related outputs observed during development, stress, and senescence depend in part on variation in pathway-gene expression, enzyme activity, and metabolic interactions [95]. Specialization within multigene families determines which enzymes are available in particular organs or developmental stages. Transcriptional, chromatin, and post-transcriptional control further adjust their abundance, whereas post-translational modification, enzyme stability, and protein interactions influence activity and substrate use [96]. These regulatory processes provide potential mechanisms for the pathway changes discussed above, although their quantitative contributions to metabolic flux remain to be established.

### 5.1. Gene Specialization and Expression Control

Many carotenoid pathway enzymes are encoded by multigene families whose members differ in tissue distribution, developmental timing, and stress responsiveness [97]. The PSY family provides a clear example. In tomato, *PSY1* mainly supports carotenoid accumulation in chromoplast-rich fruits and flowers, whereas *PSY2* has a stronger role in leaf photoprotection and also contributes to seed dormancy and root responses to salinity. *PSY3* is preferentially expressed in roots and is associated with SL biosynthesis and mycorrhizal interactions [98]. Saffron *PSY* genes similarly display distinct spatial and stress-responsive expression patterns, with *CsPSY1b* responding to light stress and *CsPSY1a*, *CsPSY1b*, and *CsPSY3* showing differential induction in roots under environmental stress [99].

Functional differences within the PSY family also reflect evolutionary divergence. Monocot *PSY3* genes are generally associated with stress-induced root carotenogenesis and ABA production, whereas dicot *PSY3* genes form a distinct lineage more closely linked to root responses to nutrient status; related functions in monocots may be partly compensated by *PSY2* [100]. Regulatory diversity can also arise from a single-copy gene. *Arabidopsis PSY* produces alternative 5′ untranslated region isoforms, with the shorter isoform preferentially induced under stress and associated with rapid adjustment of carotenoid flux [101].

Comparable specialization occurs at downstream branches. In *Arabidopsis*, *NCED2* and *NCED3* contribute mainly to ABA accumulation and stress responses in vegetative tissues, whereas *NCED5*, *NCED6*, and *NCED9* have stronger roles in seed dormancy. *NCED5* can also cooperate with *NCED3* during vegetative water-stress responses [102]. Developmental stage can further modify the relative contribution of family members. In tetraploid wheat, *BCH2* plays a prominent role in salt responses at the seedling stage but contributes less at tillering, whereas simultaneous disruption of *BCH1* and *BCH2* causes pronounced salt hypersensitivity [103]. Functional specialization and partial redundancy therefore allow carotenoid metabolism to be adjusted without requiring uniform regulation of all pathway genes.

**Figure 4:**
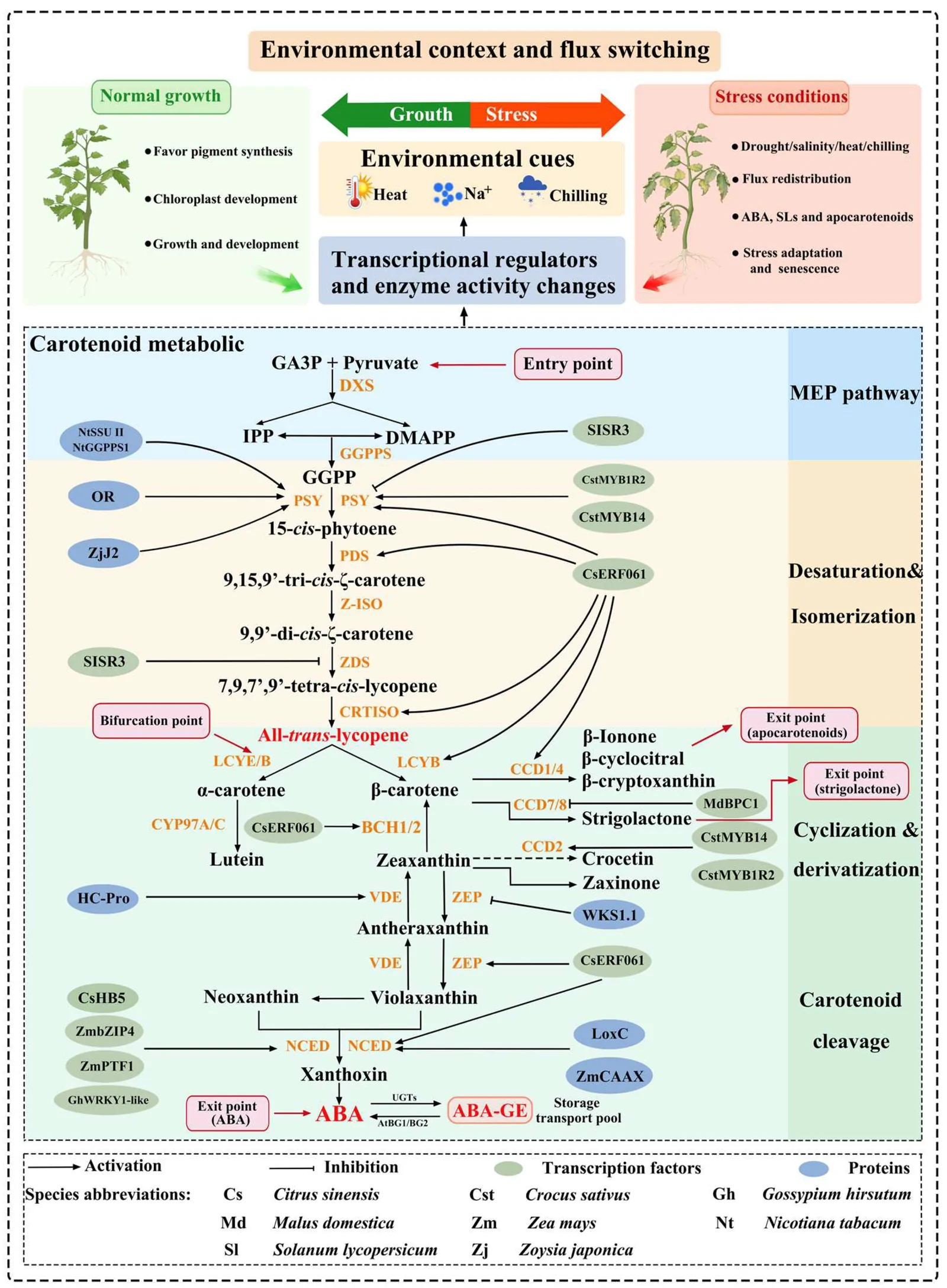
Carotenoid pathway architecture and multilevel regulation of metabolism. This pathway is regulated by environmental context and flux switching. Normal growth favors pigment synthesis and chloroplast development, while stress conditions (drought, salinity, heat, chilling) trigger flux redistribution and promote the accumulation of ABA, SLs, and apocarotenoids, thereby facilitating stress adaptation and senescence. These cues are integrated through transcriptional regulators and enzyme activity changes. The pathway is divided into three major regions according to metabolic function. The methylerythritol 4-phosphate (MEP) pathway supplies isopentenyl diphosphate (IPP) and dimethylallyl diphosphate (DMAPP), which are used by geranylgeranyl diphosphate synthase (GGPPS) to generate GGPP. During desaturation and isomerization, PSY converts GGPP to phytoene, which is subsequently converted to all-*trans*-lycopene through reactions catalyzed by PDS, Z-ISO, ZDS, and CRTISO. During cyclization, derivatization, and cleavage, lycopene is partitioned into the α- and β-carotene branches and subsequently converted into xanthophylls, ABA precursors, strigolactones, zaxinone, and other carotenoid-derived metabolites. Representative transcription factors and regulatory proteins that activate or inhibit individual pathway steps are positioned adjacent to their reported targets. Green ellipses indicate transcription factors and blue ellipses indicate regulatory proteins. Arrowheads indicate positive regulation, whereas blunt-ended lines indicate negative regulation. ABA, abscisic acid; BCH, β-carotene hydroxylase; CCD, carotenoid cleavage dioxygenase; CRTISO, carotenoid isomerase; DMAPP, dimethylallyl diphosphate; DXS, 1-deoxy-D-xylulose 5-phosphate synthase; GA3P, glyceraldehyde 3-phosphate; GGPP, geranylgeranyl diphosphate; GGPPS, geranylgeranyl diphosphate synthase; IPP, isopentenyl diphosphate; LCYB, lycopene β-cyclase; LCYE, lycopene ε-cyclase; NCED, 9-*cis*-epoxycarotenoid dioxygenase; PDS, phytoene desaturase; PSY, phytoene synthase; SLs, strigolactones; VDE, violaxanthin de-epoxidase; ZAS, zaxinone synthase; ZDS, ζ-carotene desaturase; ZEP, zeaxanthin epoxidase; and Z-ISO, ζ-carotene isomerase. In the figure, the green arrow (pointing to the lower right) and the red arrow (pointing to the lower left) represent the plant responses under normal and stress conditions, respectively, both pointing to the changes in transcriptional regulators and enzyme activity.

The activity of these specialized genes is further shaped by stress-responsive transcription factors. *WRKY*, *MYB*, *bZIP*, *AP2/ERF,* and homeobox proteins can regulate individual pathway steps or coordinate several metabolic nodes simultaneously [104]. In citrus, *CsERF061* activates genes involved in both carotenoid biosynthesis and cleavage, including *PSY1*, *PDS*, *CRTISO*, *LCYB1*, *BCH*, *ZEP*, *NCED3*, *CCD1*, and *CCD4* [105]. In saffron, *CstMYB1R2* and *CstMYB14* regulate *PSY* and *CCD2*, linking transcriptional control with carotenoid and apocarotenoid production [106,107]. Repression can redirect flux in the opposite direction: salt stress reduces tomato *SlSR3* expression and releases its repression of *PSY2* and *ZDS*, whereas apple *MdBPC1* represses *CCD7* under drought and waterlogging [84,108] (Figure 4).

Transcriptional regulation of *NCED* genes is particularly relevant because it directly affects ABA biosynthetic capacity. *GhWRKY1-like*, *ZmPTF1, ZmbZIP4*, and *CsHB5* activate different NCED family members and are associated with enhanced drought or cold responses through increased ABA accumulation [90,109,110,111]. However, excessive activation of ABA biosynthesis can impose substantial growth costs. Strong constitutive expression of *LeNCED1* causes chlorophyll loss, photobleaching, and severe growth inhibition, whereas more moderate increases in ABA can improve water-use efficiency without comparable penalties [112]. The effect of transcriptional regulation therefore depends on both the identity of the regulated gene and the magnitude of pathway activation (Figure 4).

**Figure 5:**
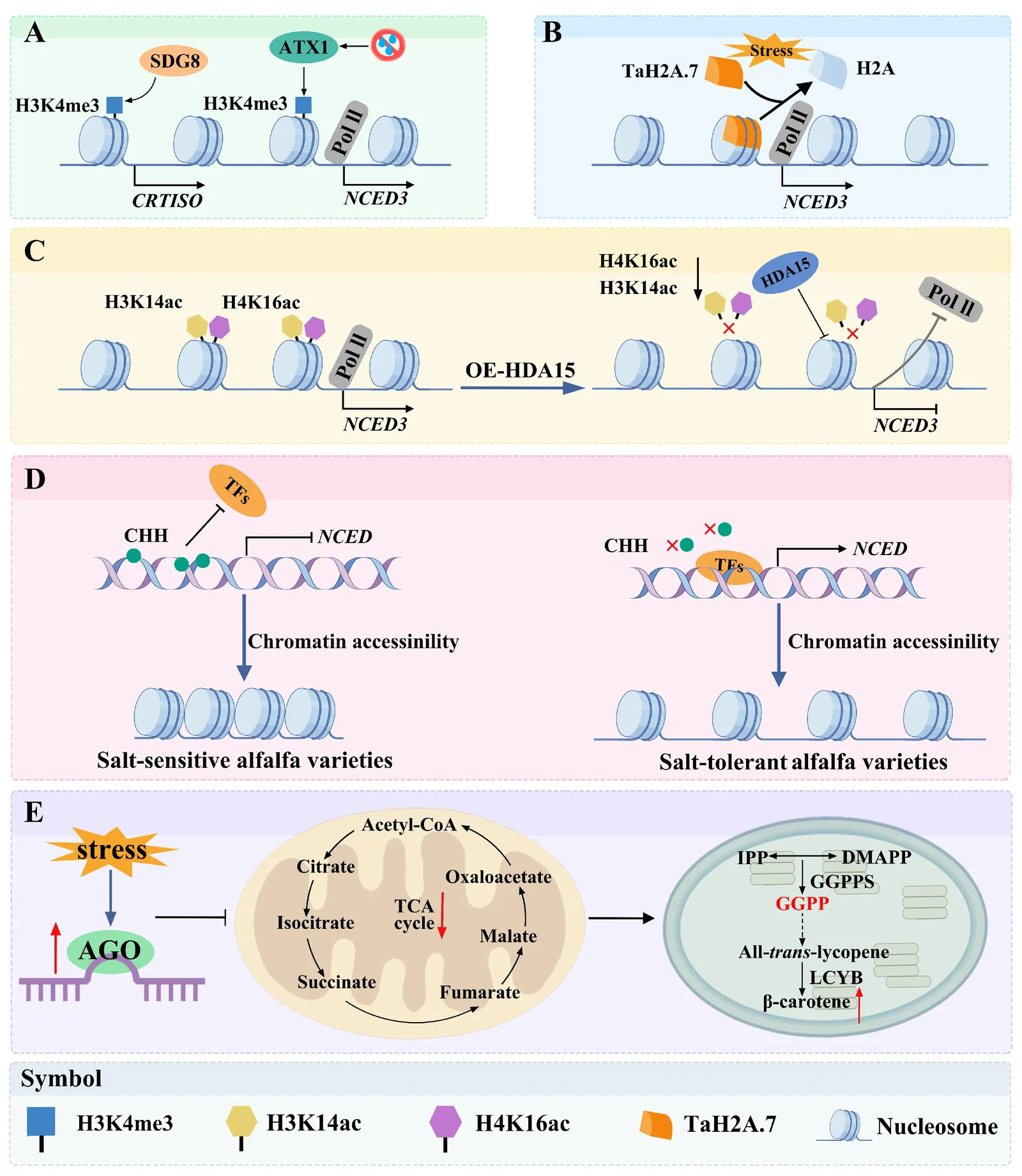
Chromatin and post-transcriptional regulation of carotenoid pathway genes. Chromatin modifications, histone variants, DNA methylation, and miRNA-mediated regulation contribute to stress-responsive control of carotenoid pathway genes. (**A**) In *Arabidopsis*, the histone methyltransferase SDG8 maintains H3K4me3 at the CRTISO locus and promotes its transcription. During dehydration, ATX1 promotes H3K4me3 deposition at NCED3 and facilitates RNA polymerase II recruitment, thereby increasing NCED3 transcription and ABA biosynthetic capacity. (**B**) In wheat, the stress-responsive histone variant TaH2A.7 replaces canonical H2A and alters nucleosome stability, promoting transcription of stress-responsive genes including NCED3. (**C**) HDA15-dependent histone deacetylation reduces H3K14ac and H4K16ac at the NCED3 locus, thereby restricting NCED3 transcription and excessive ABA accumulation under salinity. (**D**) In alfalfa, reduced CHH methylation in the NCED promoter is associated with increased chromatin accessibility and transcription factor binding, resulting in enhanced NCED expression and greater salt–alkali tolerance. (**E**) In *Dunaliella salina*, stress-induced novel-m0533-3p is associated with changes in central carbon metabolism and increased β-carotene accumulation, consistent with enhanced carbon allocation toward GGPP and carotenoid biosynthesis. H3K4me3, trimethylation of histone H3 lysine 4; H3K14ac, acetylation of histone H3 lysine 14; H4K16ac, acetylation of histone H4 lysine 16; GGPP, geranylgeranyl diphosphate; and Pol II, RNA polymerase II. In the figure, solid arrows indicate positive regulation, promotion, or metabolic flow, whereas blunt-ended lines indicate negative regulation or inhibition.

Chromatin and post-transcriptional mechanisms provide additional control over stress-responsive expression. In *Arabidopsis*, SDG8 maintains H3K4me3 at CRTISO and promotes its transcription, thereby affecting lutein accumulation and downstream carotenoid metabolism [113]. During dehydration, ATX1 is recruited to NCED3 and promotes H3K4me3 deposition and RNA polymerase II recruitment [114] (Figure 5A), whereas HDA15 modifies histone acetylation at NCED3 and limits excessive ABA accumulation under salinity [115] (Figure 5C). The wheat histone variant TaH2A.7 also affects NCED3 expression under drought [116] (Figure 5B), while reduced CHH methylation (H = A, C, or T) in the NCED promoter is associated with increased chromatin accessibility, enhanced NCED expression, and improved salt–alkali tolerance in alfalfa (*Medicago sativa* L.) [117] (Figure 5D). At the post-transcriptional level, stress-responsive miRNAs target carotenoid cleavage oxygenase genes in rice [118], and the stress-induced miRNA novel-m0533-3p in *Dunaliella salina* is associated with β-carotene accumulation and changes in pathways associated with GGPP supply [119] (Figure 5E). These regulatory processes provide complementary routes for adjusting pathway-gene expression before changes in enzyme activity occur.

### 5.2. Post-Translational Regulation and Enzyme Interactions

Carotenoid flux can also be adjusted directly at the protein level through changes in enzyme activity, stability, and turnover. Depending on the target enzyme, such regulation can influence abiotic stress-related processes such as photoprotection and ABA-associated responses, as well as biotic stress-related defense against pathogens. In wheat, WKS1.1 phosphorylates and suppresses ZEP1, altering xanthophyll-cycle activity and contributing to stripe rust resistance [120]. In maize, ZmCAAX promotes degradation of ZmNCED3 and restricts ABA accumulation. Loss of this regulation enhances drought resistance but also causes chloroplast degradation, reduced photosynthetic efficiency, and lower grain yield [121], illustrating the need to constrain stress-induced metabolic responses when they become detrimental to growth (Figure 4).

Protein stability can also be promoted rather than reduced. Orange (OR) proteins in sweet potato (*Ipomoea batatas)* and *Arabidopsis* interact directly with PSY and protect the enzyme from degradation without requiring major changes in PSY transcription [122] (Figure 4). The naturally occurring IbOR-R96H variant further enhances carotenoid accumulation and is associated with improved salt and heat tolerance [123]. OR-mediated stabilization can thus modify carotenoid biosynthetic capacity independently of major changes in PSY transcript abundance.

Protein–protein interactions further influence how metabolic substrates are distributed. In tobacco, NtSSU II forms a heterodimer with NtGGPPS1 and interacts with NtPSY1, generating a complex that promotes channeling of GGPP toward carotenoid biosynthesis under high light [124] (Figure 4). Physical association between precursor-producing and precursor-consuming enzymes can therefore increase the efficiency with which GGPP is directed toward selected metabolic products, particularly under changing environmental conditions.

Other interactions connect carotenoid enzymes with broader stress-response pathways. Tomato SlNCED2 interacts with lipoxygenase LOXC, linking ABA metabolism with oxylipin-associated drought responses [125]. Maize ZmVDE interacts with the viral RNA-silencing suppressor HC-Pro, indicating that a xanthophyll-cycle enzyme can also participate in antiviral defense [126]. In *Zoysia japonica*, the interaction between ZjJ2 and ZjPSY links PSY stability with ABA-related responses [127] (Figure 4). These interactions connect carotenoid metabolism with enzyme stability, substrate channeling, and stress-associated processes beyond pigment biosynthesis. These effects are further shaped by metabolite feedback and competition for shared precursors, which are discussed in the following section.

## 6. Metabolic Feedback and Resource Allocation in Carotenoid Networks

Carotenoid pathway activity is also influenced by the metabolic state of the pathway itself. Changes in the abundance of intermediates can feed back on gene expression, protein accumulation, or downstream branch activity, while variation in precursor supply can alter how carbon is distributed among carotenoids and other plastidial isoprenoids [128]. Metabolite feedback and precursor competition therefore provide an additional level of control over carotenoid flux, allowing pathway output to adjust to changing metabolic and environmental conditions.

### 6.1. Metabolite Feedback and Metabolic Signaling

Carotenoid intermediates can influence both upstream pathway activity and downstream branch allocation. In tomato, the *yellow-flesh* (*r*) mutation abolishes *PSY1* transcription and blocks carotenoid biosynthesis, whereas introduction of the *tangerine* (*t*) mutation leads to the accumulation of *cis*-carotenoids such as prolycopene and partially restores *PSY1* transcription [129]. Conversely, depletion of these *cis*-intermediates through heterologous expression of bacterial crtI reduces PSY protein abundance and phytoene production. Changes further downstream can also alter pathway output: accumulation of all-*trans*-lycopene following *crtI* expression induces carotenoid cyclase genes and favors flux toward the β-carotene branch [130]. These findings indicate that carotenoid intermediates can influence pathway activity at both transcriptional and protein levels and may contribute to redistribution of precursor use among downstream branches.

Carotenoid-derived oxidation products provide a related but functionally distinct form of metabolic regulation by linking pathway turnover with stress signaling. β-CC, generated from β-carotene oxidation under high light, activates singlet-oxygen-responsive transcription and participates in plastid-to-nucleus communication [9]. In this way, oxidative modification of carotenoid pools can generate signals that report changes in chloroplast redox status and modify downstream gene expression. Carotenoid metabolism therefore responds to environmental signals while also producing metabolites that can feed information back into transcriptional and stress-response pathways. However, the magnitude and direction of these responses depend on the availability of precursors entering the pathway.

### 6.2. Precursor Competition and Metabolic Prioritization

The MEP pathway supplies IPP and DMAPP for plastidial isoprenoid biosynthesis, making precursor availability an important constraint on carotenoid production under stress [97]. In *Picea glauca*, drought strongly reduces photosynthetic carbon assimilation and MEP pathway flux while carotenoid levels remain comparatively stable, suggesting preferential maintenance of selected isoprenoid end products under limited carbon supply [131]. In *Haematococcus pluvialis*, stress-responsive expression of *DXS*, *CMS*, *CMK*, *HDS*, and *HDR* is associated with carotenoid accumulation under high light and nitrogen starvation [132], whereas overexpression of *PmDXR* in *Arabidopsis* increases DXR activity and carotenoid accumulation [133]. These observations associate precursor-pathway regulation with changes in carotenoid abundance, although most do not directly quantify carotenoid-specific synthesis rates.

Competition for precursor carbon becomes particularly apparent at the GGPP node. GGPP is shared by carotenoid biosynthesis and other plastidial isoprenoid pathways, including those producing chlorophyll-associated isoprenoids, tocopherols, and gibberellins. GGPPS therefore lies at an important branch point for allocating precursor carbon among these products [134]. In *Arabidopsis*, *AtGGPPS11* supplies GGPP to photosynthesis-related isoprenoid metabolism and interacts with *PSY* to support carotenoid biosynthesis [135]. In tobacco, formation of the NtSSU II–NtGGPPS1–NtPSY1 complex promotes GGPP channeling toward carotenoid synthesis under high light [124]. By contrast, OsGRP recruits *OsGGPPS1* into a chloroplast complex associated with chlorophyll biosynthesis in rice. Overexpression of *OsGRP* increases chlorophyll content while decreasing carotenoid and gibberellin accumulation, consistent with preferential allocation of GGPP toward the chlorophyll branch [136]. In drought-tolerant wild tomato, higher expression of *GGPPS* and *HPT1* is associated with increased α-tocopherol accumulation, consistent with increased investment in tocopherol biosynthesis during drought adaptation [137].

The distribution of shared isoprenoid precursors is therefore a potential control point for balancing carotenoid production with chlorophyll, tocopherol, and hormone-associated metabolism. Tissue-specific expression, enzyme interactions, and substrate channeling may favor particular branches under different conditions, but changes in end-product abundance should not be equated with measured carbon flux. Identifying the conditions under which these mechanisms alter pathway rates remains an important objective for stress biology.

## 7. Conclusions and Perspectives

Carotenoid metabolism contributes to plant adaptation through photoprotection, redox-associated responses, and the formation of hormonal and other signaling-active metabolites. Evidence discussed here connects changes in precursor supply, biosynthetic enzymes, branching reactions, xanthophyll cycling, and cleavage with distinct stress-related outcomes. Gene-family specialization and regulation at transcriptional, chromatin, RNA, and protein levels further influence pathway activity, while metabolic intermediates and shared precursors can affect pathway balance. Comparisons across abiotic stress, biotic challenges, and senescence extend this view to different environmental and developmental conditions. A central limitation remains that many reported changes concern gene expression or metabolite abundance rather than direct measurements of pathway flux.

A major unresolved question is whether, and to what extent, carotenoid flux is quantitatively redistributed under different environmental stresses and during senescence. The MEP pathway and the GGPP node supply precursors to several plastidial isoprenoid branches, including carotenoids, chlorophyll-associated metabolites, tocopherols, and gibberellins. Current studies document changes in precursor-pathway expression, enzyme activity, and metabolite pools, but they rarely resolve rates of carbon entry and allocation across competing branches. Stable-isotope tracing and turnover measurements, complemented by time-resolved metabolomics and enzyme-activity analyses, will be needed to test proposed changes in branch allocation. Particular attention should be given to whether stress adaptation involves increased overall precursor production or preferential allocation of limited precursor pools toward specific carotenoid and non-carotenoid branches.

The mechanisms that establish spatial and temporal specificity in carotenoid metabolism also remain incompletely understood. Functional differences and partial redundancy among PSY, NCED, BCH, and other gene families provide plants with considerable metabolic flexibility, yet the factors that determine which family member predominates in a particular tissue or stress condition are still poorly defined. Transcription factors, chromatin modifications, alternative transcripts, small RNAs, protein turnover, and enzyme interactions can all contribute to this specificity, but their relative importance and coordination remain largely unresolved. Determining how these regulatory processes converge on individual pathway nodes will be important for explaining why manipulation of the same carotenoid gene can produce different physiological outcomes among tissues, developmental stages, species, and environmental conditions.

Another important direction concerns the perception and downstream action of carotenoid-derived signals. ABA and SLs have well-characterized receptors and signaling components, whereas the molecular mechanisms through which zaxinone, β-ionone, β-CC, and related apocarotenoids exert their biological effects remain much less defined. Their production is often closely associated with changes in ROS, chloroplast redox status, hormone metabolism, and retrograde signaling, making it difficult to distinguish direct signaling functions from secondary consequences of altered carotenoid metabolism. Identifying their molecular targets, defining their effective concentrations and sites of action, and determining how their signals interact with established hormone and redox pathways will be necessary to understand how carotenoid turnover is translated into specific stress responses.

Translating this knowledge into crop improvement will require greater attention to metabolic balance. Increasing carotenoid or ABA abundance can improve stress tolerance in some settings, but constitutive pathway activation or altered ABA homeostasis may also impair photosynthesis, development, or yield. Future engineering strategies should therefore aim to control where, when, and to what extent carotenoid flux is modified rather than simply maximizing carotenoid accumulation. Tissue-specific or stress-inducible manipulation of selected pathway nodes, combined with direct assessment of metabolic flux, active hormone availability, and field performance, may offer a more effective route to improving stress resilience while minimizing growth penalties. A clearer understanding of carotenoid flux, regulatory specificity, signaling mechanisms, and metabolic trade-offs should ultimately enable more precise manipulation of carotenoid metabolism to coordinate photosynthetic performance, growth, and stress adaptation.

## Data Availability

No new data were created or analyzed in this study. Data sharing is not applicable to this article.
